# Do students achieve the desired learning goals using open-book formative assessments?

**DOI:** 10.5116/ijme.5bc6.fead

**Published:** 2018-11-19

**Authors:** Stefan P. Minder, David Weibel, Bartholomäus Wissmath, Felix M. Schmitz

**Affiliations:** 1 Dean's Office, Medical Faculty of the University of Bern, Switzerland; 2 Department of Psychology, University of Bern, Switzerland; 3 w hoch 2 GmbH, Bern, Switzerland; 4 Institute of Medical Education, Medical Faculty of the University of Bern, Switzerland

**Keywords:** Formative assessment, course preparation, blended learning, medical curriculum, practical course

## Abstract

**Objectives:**

The present study aimed to examine whether medical students benefit from an open-book online formative assessment as a preparation for a practical course.

**Methods:**

A between-subjects experimental design was used: participants – a whole cohort of second-year medical students (N=232) – were randomly assigned to either a formative assessment that covered the topic of a subsequent practical course (treatment condition) or a formative assessment that did not cover the topic of the subsequent course (control condition). Course-script-knowledge, as well as additional in-depth-knowledge, was assessed.

**Results:**

Students in the treatment condition had better course-script knowledge, both at the beginning, t_(212)_ = 4.96, p < .01, d = 0.72., and in the end of the practical course , t_(208)_ = 4.80, p < .01, d = 0.68. Analyses of covariance show that this effect is stronger for those students who understood the feedback that was presented within the formative assessment, F_(1, 213)_=10.17, p<.01. Additionally, the gain of in-depth-knowledge was significantly higher for students in the treatment condition compared to students in the control condition, t_(208)_ = 3.68., p < .05, d = 0.72 (0.51).

**Conclusions:**

Students benefit from a formative assessment that is related to and takes place before a subsequent practical course. They have a better understanding of the topic and gain more in-depth-knowledge that goes beyond the content of the script. Moreover, the study points out the importance of feedback pages in formative assessments.

## Introduction

The purpose of formative assessment (FA) is to support learning, as opposed to a summative assessment, where the purpose is validation and accreditation.^1^ Frequently, an online FA implicitly assumes a closed-book situation with an emphasis on the importance of the score achieved as a formative element.^2^^, ^^3^^, ^^4^ Learning is promoted with the use of such FAs by enabling the students to recognize and close gaps.^5^ If the FA is viewed as a holistic process, it is followed by feedback given to the students and possibly, changes in the tuition.^6^ Arnold^7^ reports that cheating in closed-book FA is disadvantageous to students. However, an online FA often takes place in an unproctored environment (for example, at home^8^). There is a clear lack of research that investigates open-book FAs. To our knowledge, only one empirical study has been conducted to examine this issue: In 2006, Krasneand colleagues.^9^ compared both FA modalities (open-book and closed-book) and found the open-book variant to enhance higher order reasoning, including the ability to identify and access appropriate resources and to integrate and apply knowledge. Compared to the closed-book variant, the open-book FA was a better predictor for the students’ exam score. Therefore, the study of Krasne and colleagues.^9^ indicates that the performance assessed with open-book FA (notwithstanding the use of resources), can remain a formative statement about students without the cheating disadvantage described by Arnold.^7^

Previous studies point out that according to lecturers of practical courses, the preparation that is based on the course script, together with a successful performance of the practical exercises, is essential in acquiring in-depth-knowledge of medical and scientific contexts during the practical course. The cognitive load theory provides arguments supporting this assumption.^10^^, ^^11^

If during the course, the students use most of their mental resources for procedural tasks (intrinsic cognitive load) while continually consulting the course script (extraneous cognitive load), the remaining mental capacity to build more in-depth-knowledge (germane cognitive load) becomes too low. According to the lecturers, the cause of poorly prepared students could be the low coverage of the course content in upcoming summative examinations. A survey revealed that medical students stressed the importance of learning relevant content for upcoming examinations.^12^ A steering effect of exams on students’ learning behavior was discussed by Albanese and colleagues.^13^, and according to Heenemann and colleagues.^14^, this also applies to FA; the influence of both formative and summative assessment could even have a negative influence on learning behavior. Therefore, Heenemann and colleagues.^14^ claim that an assessment strategy for the whole curriculum is needed (Programmatic Assessment). Wood^15^ notes that students need support in the planning of learning activities with respect to time management and setting content priorities. Self-directed learning is typically a large part of the study activities in the context of a Problem Based Learning (PBL) curriculum. Wood states that, “Students may be unsure how much self-directed study to do and what information is relevant and useful”.^15^ With regard to time management, our intention intervention was to provide students with a structured learning sequence of 30 to 60 minutes as homework, therefore promoting self-directed learning, as described by Clark.^16^

In our study, we examined whether open-book online FA is a useful preparation for compulsory practical courses in a bachelor's medical curriculum. The purpose of our study was to empirically test whether medical students benefit from an open-book online FA as preparation for a practical course. We investigated whether an open-book online FA as a preparation for practical courses has a positive impact on course-script-knowledge and additional in-depth-knowledge. Course-script knowledge is essential, as it enables the students to fulfill the tasks in the practical course. The construct "in-depth knowledge" addresses the didactic goals of the lecturer: Students should be able to solve problems that are not explicitly highlighted during the course. They should achieve this by deriving knowledge patterns, defined by the lecturers from course insights.

Critics of FA point out lacking evidence that FA promotes learning.^17^ In open-book online FA, we expect that learning is facilitated, on the one hand, when students answer the K-type multiple-choice questions (MCQ) while researching the course script and other sources, and, on the other hand, when reading the feedback page (solutions, explanations, score) that is displayed after they have entered their choice. We assessed this learning gain at the beginning and the end of a 4-hour practical course and examined the additional in-depth-knowledge gained during the course.

## Methods

### Participants

Instead of testing a sample of participants, a whole cohort of undergraduate medical students studying at the University of Bern took part in the study (N = 232). Sixty percent of the participants were female, 40 percent were male, and the mean age was 22 years. All of them were in their second academic year. Participation in the study was voluntary. All participants were debriefed during a lecture following the practical course. Furthermore, all participants were able to access the FA used in the study, so that upcoming exam would not be affected. All participants were treated according to the Declaration of Helsinki, and the study was approved by the research ethics committee of the Canton of Bern.

### Design

We conducted a field experiment, whereby one variable was manipulated between participants. The experiment took part in the context of the practical course “Performance Physiology.” Participants were randomly assigned to either the treatment condition (a relevant FA) or a control condition (an irrelevant FA). In the treatment condition, participants completed an FA that covered the topic of the subsequent practical course, “Performance Physiology.” In the control condition, participants also completed an FA, however, the content of the control FA was not related to performance physiology. Participants did not know whether they were in the control or treatment condition.

Several performance measures for performance physiology, subjective measures, and measures concerning the completion of the FA were included as dependent variables. We measured course-script knowledge on performance physiology three times: within the open-book online FA (course-script knowledge t1), right before the practical course (course-script-knowledge t2), and right after the practical course (course-script-knowledge t3). As a fourth performance measure, we assessed the gain of additional in-depth-knowledge. Prior to the course, we further assessed whether the students were motivated to take part in the course (motivation). After the course, the satisfaction with the practical course, the subjective relevance of the practical course, as well as the relevance of the FA were examined. Additionally, we analyzed the time that was spent to complete the FA (duration of completion), the time-point to complete the FA (date of submission), and the self-perceived judgment of whether the provided feedback pages within the FA were understood (understanding of feedback). We considered the use of aids during the FA and, as a control variable, the mean grade of the last three exams. Furthermore, the teaching staff rated the quality of the students’ participation in each practical course. They were asked whether the students performed well in the course (students’ performance) and whether the students were well-prepared for the course (students’ preparation).

### Procedure

All participants were sent an invitation to participate in the FA. This was either the FA on the subject of performance physiology (treatment condition) or the control FA. Before the FA could be completed, the course-script-knowledge on performance physiology was assessed (baseline measurement). The FA had to be completed before the practical course took place. Before the start of the practical course, the knowledge was tested again. At the end of the course, the course-script-Knowledge was again evaluated. In-depth knowledge on performance physiology was also measured. The procedure of the study is described in detail below.

#### Description of the open-book online formative assessment

The learning management system of the Medical Faculty of the University of Bern served as a platform to offer two different FAs, both explicitly declared as being open-book, and included the download link to the respective course script. The first FA was designed to prepare the students for the upcoming practical course, “Performance Physiology”, a course that appears in many medical curricula around the world. The second FA consisted of questions concerning human anatomy. This second FA served as a control condition since all subsequent measurements (e.g. course-script-knowledge) were about performance physiology. Thus, the questions on human anatomy were irrelevant for the subsequent tests. Both FAs consisted of 20 multiple-choice questions with a total of 84 statements to be assessed (K-type questions). For each statement, the students had to indicate whether it was true or false. Each multiple-choice question, with the corresponding statements, was presented on one screen (question page), and all statements had to be judged before the student could continue. Each question page was followed by a feedback page that displayed the question, the statements, and the student’s previous answers. In addition, there was a detailed explanation for every statement explaining why it was true or false. At the bottom of each feedback page, the student rated his or her feedback-understanding on a four-point scale. Then, the student clicked the submit button that took them to the next question page. Backward navigation was not possible. Unique access was ensured through a personal login code. In case of an interruption, the system resumed at the last page visited. The questions in the treatment FA were based strictly on the content of the official course script, and they had been developed by a didactical expert and reviewed by lecturers. Usage duration per question page and per feedback page and timestamps were recorded. The last feedback page was followed by a short one-screen questionnaire to evaluate the just-completed open-book online FA. For the completion of the FA, the students needed between 11 to 202 minutes (mean=32 minutes; median=29 minutes).

#### Group assignment

For the practical course “Performance Physiology”, the students had been randomly placed into five groups of 45-48 individuals. The faculty’s timetable gave the odd number of groups. Each group participated once in the four-hour practical course “Performance Physiology.” Of the five groups, three had been randomly assigned to the treatment condition and two to the control condition. The content of the practical course was identical for all five groups. The students, as well as the teaching staff, were blind as to the experimental condition of the groups. The difference between treatment and control groups was the topic of questions in the FA. Students in the treatment condition answered questions that were relevant for the practical course “Performance Physiology”. Students in the control FA answered questions on human anatomy that were not relevant for their internship specialization.

#### Invitation to participate in the open-book online formative assessment

For this study, 232 second-year medical students (95 in the control and 137 in the treatment condition) were invited via email to participate in the FA. They were unaware of the fact that this study was conducted in the context of the course “Performance Physiology.” Access to the FA was granted through a link in the invitation email that was sent ten days before the course. When the students clicked on the link, they saw, depending upon their personal login, either an FA for the practical course “Performance Physiology”, or questions on “Human Anatomy,” if they belonged to the control group. The text in the email explained that all records would be stored anonymously and that the performance in the FA would not have any further impact on the course of their studies. Three days before the class, a reminder was sent to the students who had not yet completed the FA or not yet clicked on the link. Sending invitations through email to fill out online questionnaires of various topics is a regular practice at our faculty. In general, participation is high, which is useful for the current study.

#### Conducting the practical course “Performance Physiology”

The purpose of this course was to provide hands-on experience in performance physiology and to learn practical skills.The practical course “Performance Physiology” ran the same way for all groups in terms of organization and structure. Identical teaching staff were involved in all five classes (one lecturer and three tutors). In the first 15 minutes of each class, all participants were asked to fill out a paper questionnaire (t2) that asked questions from the treatment FA (i.e. the students in the control group also had to respond to items related to Performance Physiology). The questionnaire also assessed the motivation to attend the course. Subsequently, the students performed the course activity (groups of three students set up physiological experiments on home trainers). In the last 15 minutes of the course, students answered another paper questionnaire (t3) with K-type multiple-choice questions for in-depth knowledge. These questions had been developed by the lecturer to represent the desired additional learning. The answers to these questions were not part of the course materials or course script and had not been discussed in class. In addition, questions concerning the course script were asked again and the self-perceived relevance and quality of the practical course were evaluated. In the end, the lecturer and his three assistants evaluated the course of the practical course. They had been naive as to whether or not a course consisting of students with the matching or mismatching FA preparation.

### Measured variables

The measured variables consisted of four performance measures and four subjective measures. The subjective measures were rated on a five-point Likert scale (1=I fully disagree; 5=I fully agree); in order to avoid response sets, several items were reverse-coded.

#### Course-script-knowledge t1 (baseline)

Before the completion of the open-book online FA, the students had to answer 20 multiple-choice questions with a total of 84 statements concerning the topic of the course script (treatment: practical course “Performance Physiology”; control: “Human Anatomy”). Each statement had to be judged as true or false (example statement: “The hematocrit is usually about 60% in healthy humans”). Correct answers gave 1 point, wrong answers -1 point. As a score, the mean values of all answers were included. Thus, possible mean values ranged from -1 to 1. The questions covered the topic of the FA.

#### Course-script-knowledge t2

In the first 15 minutes of the practical course, all students (treatment and control) had to answer five multiple-choice questions with a total of 25 statements concerning the course script of the practical course “Performance Physiology” (MCQ K-type as in t1, same score computation, but paper-based and closed-book).

#### Course-script-knowledge t3

In the last 15 minutes of the practical course, the students had to answer five multiple-choice questions again with a total of 25 statements concerning the course script of the practical course. The procedure was the same as at t2.

#### Gain of additional in-depth-knowledge

Also at t3 (in the last 15 minutes of the practical course), the students had to answer five additional multiple-choice questions with a total of 25 statements. All questions addressed additional in-depth-knowledge not covered by the course script. The ‘gain of additional in-depth-knowledge’ construct is based on the demand from the lecturer who designs and heads the course, that after completing the course, the students should, on the basis of the knowledge gained during the course, be able to answer further questions related to the course content correctly, but not covered in the course. In previous years (i.e. prior to these studies), the lecturers of the courses studied asked prepared, related questions orally and in writing. These questions were used in the post-test for this construct and represented the didactic goals of the lecturers. The characteristics of the questions (MCQ K-type), as well as the computation of the scores, were the same as for the other performance measures at t1 to t3.

#### Motivation

The motivation for the practical course was measured before the practical course began. The scale consisted of four items (rated from 1=I fully disagree to 5=I fully agree) (example item: “I would rather not take part in the practical course if it was not mandatory”, inverse coded). Internal consistency was sufficient (*Cronbach’s* Alpha=.63).

#### Satisfaction with the practical course

The quality of the practical course was measured after the practical course, using four items (example item: “The practical course was interesting”). Internal consistency was sufficient (*Cronbach’s* Alpha=.69).

#### Relevance of the practical course

The relevance of the practical course was measured after completion of the practical course, using six items (example item: “The practical course was relevant for my studies”). Good internal consistency resulted (*Cronbach’s* Alpha=.82).

#### Relevance of the FA

The relevance of the FA was measured after the practical course, using five items (example item: “Due to the FA, I learned new facts”). Good internal consistency resulted (*Cronbach’s* Alpha=.92).

#### Date of submission

We assessed when the FA was submitted. The variable was operationalized as amount of days before the practical course.

#### Duration of completion

The time that each student spent on each page of the FA was measured in seconds. For our analyses, we used the average time that a student spent per MCQ statement.

#### Feedback-understanding

On each feedback page, the students had to indicate whether the feedback was understood. Therefore, a four-point Likert Scale was used (from 1=“I do not understand the feedback at all” to 4=“I fully understand the feedback”).

#### Use of additional material

The students had to indicate to what extent they had used additional material while doing the open-book FA. Seven possible answers were offered (1=no material at all; 2=course script; 3=lecture slides; 4=Google; 5=Wikipedia; 6=other internet sources; 7=books).

#### Mean grade of the last three exams

As a control variable to test whether the two groups differed in terms of general academic performance level, the mean grade of the last three exams, which covered the first-year content (e.g. genetics, cellular processes), was included in our analyses.

#### Teaching staff judgment of student performance in the practical course

The rating of the quality of the course consisted of seven items (example item: “The students understood the content of the course”). Internal consistency was sufficient (*Cronbach*’s Alpha = .69).

#### Teaching staff judgment of student preparation

The rating of the quality of the course was assessed using one single item “The students were well-prepared (or not well-prepared)”.

## Results

Prior to testing the effect of the FA, we ensured that the participants in the control and treatment conditions did not differ regarding their mean grade of the last three exams that covered the first-year content (e.g. genetics, cellular processes). A t-test revealed no difference, t_(__228)_=0.04, p=.97. This indicated that participants in treatment and control groups were comparable in terms of their general academic performance level. To rule out gender as a possible confounding variable, we then tested whether males and females were equally distributed in both groups and found this to be the case, *χ*²(1, N=231) =0.35, p=.56 (plus one participant with gender not declared).

To test the effect of our manipulation (treatment vs. control), we computed multiple comparisons. Independent t-tests with Bonferroni corrections were carried out with the condition as independent, and the various performance and subjective measures as dependent variables (Table 1). As expected, the course-script-knowledge at t2 and t3 were significantly higher for students in the treatment condition compared to students in the control condition. In addition, students in the treatment condition acquired significantly more additional in-depth-knowledge. Effect sizes were either medium or high. The self-perceived relevance of the FA was significantly higher for the treatment group compared to the control group (strong effect). There were no differences in terms of motivation and self-perceived relevance and satisfaction with the course between control and treatment groups.

We further tested whether the effects of condition on the four performance measures were moderated by the completion of the FA (date and duration of completion and feedback-understanding). Analyses of covariance showed that the effects were independent of the duration of completion and submission date (allp-values >.35). However, the effect of condition on course-script-knowledge at t2 was moderated by feedback-understanding, F_(__1, 213)_=10.17, p<.01. The difference between the mean values, adjusted by feedback-understanding, was still significant (M_Treatment_=0.45 vs. M_Control_=0.37). However, the effect was weaker compared to the result of the t-test mentioned above without feedback as covariance. The moderation showed that the effect of the conditions was weaker for those students who did not understand the feedback that was presented in the FA. In contrast, the effect of condition on in-depth-knowledge was independent of feedback-understanding (p=.42).

**Figure 1:**
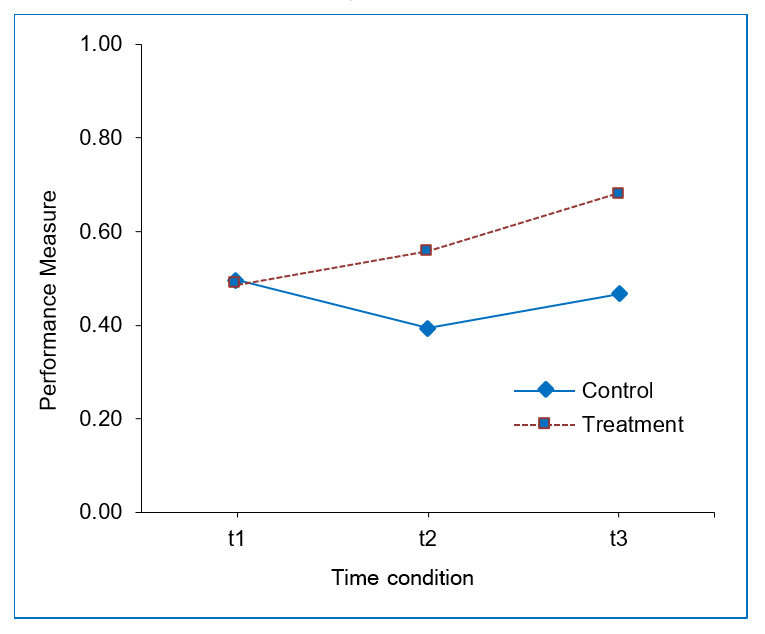
Significant interaction between the time of measurement and condition

In a next step, it was tested whether the condition influenced the three performance measures of course-script-knowledge at t1, t2, and t3. Therefore, an analysis of variance with repeated measures was computed. Time of measurement (t1 vs. t2 vs. t3) was included as a within-subjects variable and the condition (treatment vs. control) was included as a between-subjects variable. The results reveal a main effect for the time of measurement, F_(__1, 206)_=60.68, p<.01, as well as a main effect for condition (treatment vs. control), F_(1, 206)_=10.07, p<.01. Furthermore, a highly significant interaction between the two variables was found, F_(__1, 206)_=18.58, p<.01. This interaction is depicted in Figure 1. It shows that the level of course-script-knowledge continuously increases within the treatment condition, whereas the course-script-knowledge of the control group decreases from t1 to t2 and then increases from t2 to t3; the course-script-knowledge level of the treatment groups is not reached at t3.

**Table 1:** Multiple comparisons between treatment (FA) and control

Variable	Group	n	M	SD	SE	t value	Cohen’s d
Course-script- knowledge t1	control	95	.38	.31	.032	-1.61	0.20
	treatment	137	.32	.29	.025		
Course-script- knowledge t2	control	86	.31	.24	.026	4.96**	0.72
	treatment	128	.49	.26	.023		
Course-script- knowledge t3	control	87	.47	.21	.023	4.80**	0.68
	treatment	123	.61	.21	.019		
Gain of additional in-depth-knowledge	control	87	-.12	.18	.023	3.68*	0.51
	treatment	123	-.03	.17	.019		
Motivation	control	86	3.71	.55	.059	-0.25	-0.03
	treatment	128	3.69	.67	.059		
Satisfaction with Practical Course	control	87	3.32	.69	.074	0.98	0.15
	treatment	123	3.42	.69	.062		
Relevance Practical Course	control	87	3.64	.60	.064	-0.11	0.02
	treatment	123	3.65	.61	.055		
Relevance FA	control	63	1.63	.75	.094	5.94**	1.00
	treatment	85	2.39	.78	.084		

Note: *p<.05, **p< .01, two-tailed; Bonferroni corrections were carried out

Furthermore, we computed correlations between the measured variables, separately for the treatment and the control condition. *Pearson*’s correlations were computed with one exception: when duration of completion was strongly positively skewed (skewness=14.1). As a consequence, *Spearman*’s correlations were computed for this variable.

The resulting correlations are shown in Table 2. The results indicate that the variables related to the completion of FA were strongly related to performance in terms of course-script-knowledge, but not to performance in terms of additional in-depth-knowledge.

Since we offered an open-book FA, we further assessed whether students used additional material while taking part in the FA. About two-thirds of the students (65.5%) indicated that they did use some additional material. Google gathered the material that was used the most (29.3%), followed by the course script (12.9%), Wikipedia (12.5%), and other Internet sources (11.2%). Less than 10% used the lecture slides (9.5%) or books (5.2%).

In the last step, the judgment by the teaching staff concerning the performance and preparation of the students in their course were analyzed. The results show that the treatment and the control groups were not judged differently, neither in terms of performance (M_Treatment_=3.84 vs. M_Control_=4.00, t_(__23)_=-0.86, p=.40), nor in terms of preparation (M_Treatment_=3.91 vs. M_Control_=3.95, t_(23)_=0.22, p=.82).

## Discussion

We investigated whether or not open-book online formative assessment (FA), as preparation for the practical course “Performance Physiology,” is beneficial in terms of the level of course preparation and in-depth-knowledge gained during the course.

In line with our expectations, we were able to show that the use of open-book online FA, as preparation for the course, had a positive effect. In terms of course-script-knowledge, students with this preparation scored significantly higher, both at the beginning and the end of the course, than the control group. Furthermore, there was a significantly more in-depth-knowledge gain for the students in the treatment group during the course. In accordance with Krasne and colleagues.^9^, this clearly indicates that the open-book FA has a positive impact on performance in terms of the in-depth-knowledge acquisition. Additional analyses showed that this influence was not moderated by the time that was spent to complete the FA, which means that even students who spent little time on the open-book online FA experienced a learning benefit. In their FA study, Palmer and colleagues.^4^ also concluded that participation in an FA promotes learning, independent of the time spent on it.

**Table 2:** Descriptive and bivariate correlations between the measured variables

Variable	1	2	3	4	5	6	7	8	9	10	11
Treatment Group											
Performance Measures											
1. Course-script-knowledge t1	—	.52**	.32**	.14	.08	.27**	.18	.18	.74**	.68**	.77**
2. Course-script-knowledge t2		—	.61**	-.04	.12	.23*	.04	.04	.43**	.39**	.43**
3. Course-script-knowledge t3			—	.10	.08	.12	.02	-.002	.41**	.22*	.28**
4. Gain of additional in-depth-knowledge				—	-.15	.07	-.05	.09	.10	.06	.09
Self-Report on Practical Course and FA											
5. Motivation					—	.24**	.39**	.18	.09	.11	.08
6. Satisfaction with Course						—	.35**	.06	.26**	.20*	.08
7. Relevance Practical Course							—	.30**	.19	.20*	.17
8. Relevance FA								—	.29**	.07	.16
Completion FA											
9. Duration of Completion									—	.67**	.76**
10. Submission Date (amount of days before the course)										—	.76**
11. Feedback-understanding											—
Control Group											
Performance Measures											
1. Course-script-knowledge t1	—	.37**	.26*	.12	.001	-.03*	-.08	-.22	.65**	. 54**	.49**
2. Course-script-knowledge t2		—	.49**	-.05	.05	.08**	.004	-.34**	.27**	.25*	-.05
3. Course-script-knowledge t3			—	-.23*	.09	-.12	.07	-.09	.30**	.15	-.04
4. Gain of additional in-depth-knowledge				—	-.13	.02	.06	-.22	-.08		.29**
Self-Report on FP and FA											
5. Motivation					—	.25*	.33**	.21	.05	.13	.12
6. Satisfaction with Course						—	.33**	.09	-.13	.04	.02
7. Relevance Practical Course							—	.11	-.03	-.05	-.005
8. Relevance FA								—	.03	.004	-.03
Completion FA									—	.58**	.51**
9. Duration of Completion											
10. Submission Date (amount of days before the course)										—	.74**
11. Feedback-understanding											—

Note: *p<.05; **p<.01, two-tailed

The FA-based preparation did not influence the students’ motivation. Although the treatment condition resulted in better learning outcomes at the end of the course, the satisfaction with and the relevance of the course quality were not more highly rated. The teaching staff also perceived the students’ preparation and performance during the practical course as equal for both conditions. In his book, Ramsden addresses this issue: “… all aspects of University teaching should be driven by the changes in understanding we want to see to occur in our students”.^18^

Based upon cognitive load theory, we argued in the introduction that, with a lack of preparation for the practical course (that is, not consulting the course script in advance), more mental resources are used up during the course. In our study, students who prepared for the course by using the open-book online FA scored significantly better in the closed-book test at the end of the course, both for course-script-knowledge and gained in-depth-knowledge. We interpret this observation in the context of cognitive load theory in that the students had more mental resources for building up in-depth-knowledge during the course. The teaching staff perception of equal course success in both groups can be interpreted as a false conclusion because the gain of in-depth-knowledge in the control group was inadequate. It is possible that better preparation and successful gain of in-depth knowledge in the treatment condition did not result in visible behavior during the course. According to Anderson and colleagues.^19^, in-depth-knowledge is only built if the students act as active agents of the learning process: by selecting information and by forming a meaning of its own. If we apply this statement to our own on-site observations at the practical courses, the following activities could have led to the gain in in-depth-knowledge: (a) observations made during experiments, resulting in new knowledge with a meaning of its own; (b) reflection on information from  a series of lecture-style teaching during the practical course; (c) use of additional information sources during the course such as tablet PCs, books, and lecture materials,; (d) information processing during discussions among students. Regarding the last point, it should be noted that, because of good adherence to the preparatory open book online FA in the treatment group, mostly well-prepared students participated. The relatively homogeneous level of students’ preparation in each group must be seen as a positive characteristic. Burdett^20^ notes that the unequal effort of individuals that are engaged in group work is judged as the worst aspect of collaboration. If FAs were voluntary, this most likely would result in heterogeneous groups.

Based on these results, we would recommend introducing open-book online FA as preparation for practical courses. For the medical curriculum at other universities, these FAs could be useful, since it serves as an investment in the quality of the curriculum. One should consider the effort it takes to implement an FA since FAs must be created carefully. Our study cannot make exact recommendations on how open-book online FA should be designed in order to ensure the desired formative effects, although there is a script for every practical course that provides the content framework. While planning the FA for this study, it became clear that the contents had to be prioritized. The didactical expert and lecturers who were involved in the study had to anticipate the students’ knowledge gaps and formative needs. Basic concepts that were lacking or forgotten from prior studies (at the pre-university level) needed to be considered. Hunt and colleagues.^21^ also pointed out that creating good FA MCQ items is a challenge because the aim is not to have students checking the correct answers with a certain probability, but rather, the expected students’ level of knowledge should be addressed by the possible answers^21^ (including the incorrect ones). Furthermore, Hunt and colleagues.^21^ point out a need for items that highlight students’ misconceptions in order to correct them. Another important aspect to consider is the inclusion of online feedback, which has the power to establish response certitude (also called response confidence or response certainty). This increases the students’ certainty in the answer and his or her understanding. The creation of these online feedback pages in our FA followed  the guidelines of Shute. ^22^ We implemented these guidelines in the form of explanations for every correct and every incorrect answer, resulting in high editorial workload.

The results of our study emphasize the importance of feedback pages as formative agents. Feedback-understanding had a moderating effect on course-script-knowledge. Students who declared poor understanding of the feedback  in the FA acquired less course-script-knowledge. Surprisingly, this effect was not found for the gain of in-depth knowledge. In addition, low feedback-understanding was associated with late participation and short time spent on the feedback pages of the FA. Perhaps this pattern of behavior was displayed by students with poor general compliance with the curriculum’s requirements.

This study did not capture how open-book online formative assessment as a preparation for practical courses affects students’ performance during the course; it focused on the learning outcome after the course. In other words, the learning effects of the course preparation and the course itself cannot be separated and future studies should examine the impact on students' performance *during* their studies as well. If course preparation also increases student performance during their studies, this may suggest the need for changes in course design to further optimize learning outcomes with respect to the interaction between course and preparation.  Furthermore, the formative assessment was only about one topic (physiology), and its effect was only tested within one type of course. Thus, to enhance generalizability, future studies could expand this study to further topics as well.

## Conclusions

Our findings suggest that FAs can be very beneficial. The treatment FA was effective, even for students who responded late, invested only little time in the FA, and judged the FA process to be irrelevant. Our results suggest that students need to prepare adequately for practical courses in order to optimize their personal learning outcomes. In other words, students do better when they prepare and they gain more. We could show that open-book online FA as a preparation modality achieves a clear benefit on two levels: (1) knowledge of the course script content is improved and (2) the online assessment enhances students’ development of new knowledge that can be described as "in-depth-knowledge". In summary, we believe that our paper offers substantial evidence for the effectiveness of open-book online FA. Therefore, we conclude that online assessment should be included as a didactic tool in medical curricula with the aim of making course preparation a formalized and integrated part of course instruction.

### Conflict of Interest

The authors declare that they have no conflict of interest.
